# Engineering strategies for enhanced heterologous protein production by *Saccharomyces cerevisiae*

**DOI:** 10.1186/s12934-024-02299-z

**Published:** 2024-01-22

**Authors:** Meirong Zhao, Jianfan Ma, Lei Zhang, Haishan Qi

**Affiliations:** Department of Biochemical Engineering, School of Chemical Engineering and Technology, Frontier Science Center for Synthetic Biology and Key Laboratory of Systems Bioengineering (MOE), Tianjin University, Tianjin, 300350 China

**Keywords:** *Saccharomyces cerevisiae*, Protein production, Expression system, Secretion engineering, Systems metabolic engineering

## Abstract

Microbial proteins are promising substitutes for animal- and plant-based proteins. *S. cerevisiae*, a generally recognized as safe (GRAS) microorganism, has been frequently employed to generate heterologous proteins. However, constructing a universal yeast chassis for efficient protein production is still a challenge due to the varying properties of different proteins. With progress in synthetic biology, a multitude of molecular biology tools and metabolic engineering strategies have been employed to alleviate these issues. This review first analyses the advantages of protein production by *S. cerevisiae*. The most recent advances in improving heterologous protein yield are summarized and discussed in terms of protein hyperexpression systems, protein secretion engineering, glycosylation pathway engineering and systems metabolic engineering. Furthermore, the prospects for efficient and sustainable heterologous protein production by *S. cerevisiae* are also provided.

## Background

As performers and embodiments of life activities, functional proteins are important in all aspects of human life. For example, medicinal proteins, food proteins, and industrial enzymes have had important impacts on modern society [1, 2]. Medicinal proteins, as the fastest-expanding area of the global health care business, will have a global market of approximately $400 billion by 2025 [3]. The market for industrial enzymes is approximately $7 billion and growing at a 4% annual rate [4]. In addition to meeting the food protein needs of nearly 7.7 billion people worldwide, this number is increasing at a rate of approximately 1.07% each year and is predicted to reach 10 billion by 2050 [5]. As the world population is growing and the protein market is increasing, additional issues arise because traditional plant and animal proteins are unable to supply increasing protein demand. New protein products and sustainable manufacturing techniques are in high demand. Microbial production of proteins is an important strategy for alleviating this dilemma due to the flexibility and efficiency of production [5]. The microorganisms used for the production of functional proteins range from bacteria to fungi, further increasing the availability of proteins. For example, yeast has been employed for producing approximately one-sixth of all pharmaceuticals licenced for human use and is especially important in the manufacture of insulin analogues and hepatitis vaccinations [6]. Significant achievements have been made in food production through the utilization of microbial fermentation as an alternative method. Fungal single-cell proteins can serve as a direct source for producing meat alternatives, and the recombinant proteins produced by fungi can be employed as technical additives in the production of meat substitutes [7, 8]. For example, mycoprotein from *Fusarium venenatum* was used instead of chicken breast tissue to make chicken nuggets [9], the filamentous fungus *Aspergillus oryzae* was employed for the production of hamburger patties [10], and *S. cerevisiae*-derived exogenous cytokines were used to promote the growth of porcine muscle satellite cells (MuSCs) for cultured meat production [7]. In summary, microbial proteins represent a dominant paradigm for future protein manufacture.

**Fig. 1:**
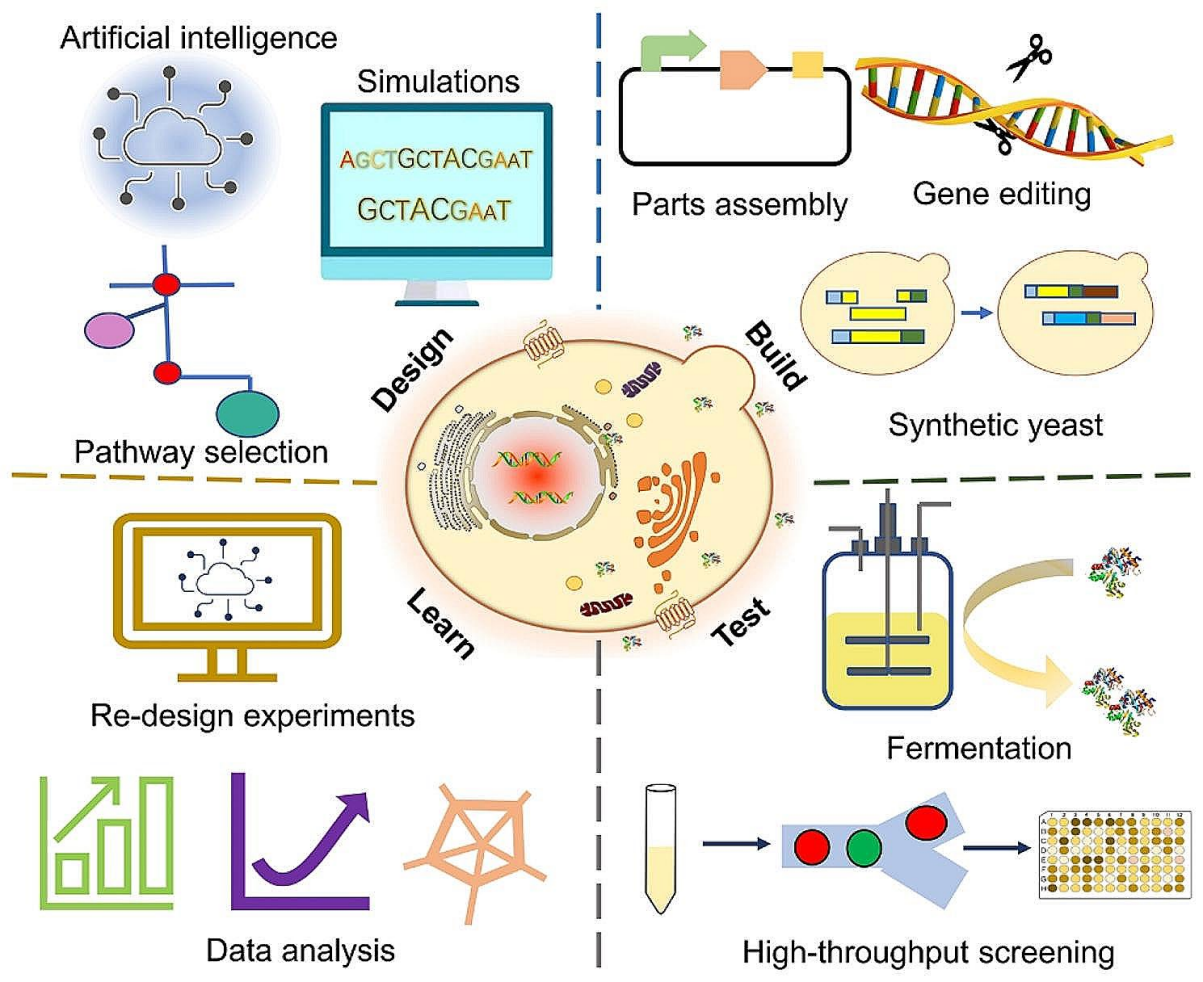
Construction of *S. cerevisiae* cell factories. The advent of new technologies has paved the way for designing *S. cerevisiae* to become a perfect production platform, significantly reducing strain construction time and accelerating the entire design, build, test, and learning cycle

Yeast is a common protein-producing host. Among microorganisms, *S. cerevisiae* is a generally recognized as safe (GRAS) microorganism. *S. cerevisiae* has a clear genetic background and an abundance of molecular biology tools that facilitate the design of strains. It is also well adapted to industrial processing and has excellent resistance to chemicals and secondary metabolites [3]. In addition, with the enrichment and refinement of metabolic engineering techniques, the “Design-Build-Test-Learn” cycle of *S. cerevisiae* has already been substantially shortened and is increasingly used for the manufacture of heterologous proteins (Fig. 1). In particular, the invention and advancement of artificial intelligence techniques have significantly improved the ability to rationally construct genetic elements, modules, and metabolic pathway networks. For example, machine learning has been used to construct promoters [11] and genome-scale metabolic models [12]. The development of metabolic models has improved the capacity to precisely control gene expression and helped forecast *S. cerevisiae* behaviour in a variety of situations [13, 14]. The efficient gene editing tool CRISPR/Cas9 has also been applied as a revolutionary and versatile strategy for genome editing in *S. cerevisiae* [15]. In addition, the *S. cerevisiae* Genome Synthesis Project (Sc2.0) intends to develop a completely synthetic yeast genome [16]. The genome resynthesis of *S. cerevisiae* enables it to have new functional and evolutionary potential and has been employed to produce valuable metabolites (alkaloids, terpenoids, flavonoids, etc.) at a high level, laying the framework for efficient protein production [17]. As research progresses, several yeasts such as *Pichia pastoris*, *Yarrowia lipolytica*, and *Kluyveromyces lactis*, have also been developed for protein production. For instance, *P. pastoris*, *Y. lipolytica*, and *K. lactis* are Crabtree negative, while *S. cerevisiae* is Crabtree positive. *P. pastoris* has a shorter mannan chain than *S. cerevisiae* [18]. In terms of substrate utilization, *P. pastoris* can utilize pentoses, glycerol and methanol as carbon sources; *Y. lipolytica* can utilize lipids; and *K. lactis* can utilize lactose [19]. *S. cerevisiae* has been modified to grow on different substrates such as glycerol [20, 21], pentose [22, 23], and methanol [24]. Although many alternative yeasts have emerged more recently, research on these alternatives has been relatively limited, and metabolic tools for these yeasts are not as rich or complete as those for *S. cerevisiae*. Therefore, *S. cerevisiae* still stands as a major workforce for recombinant protein production.

**Fig. 2:**
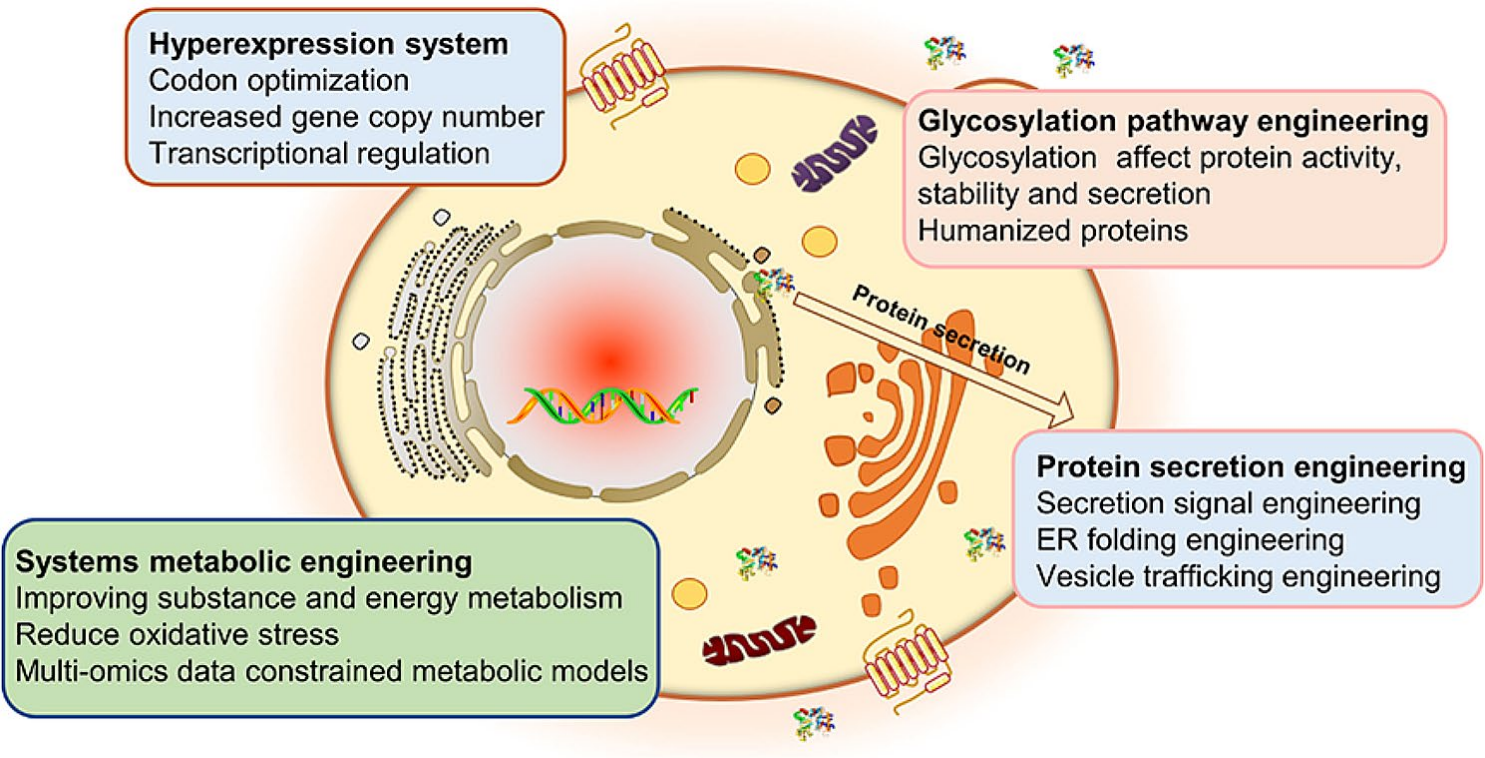
A review of engineering strategies for improved protein production by *S. cerevisiae*, including the construction of a hyperexpression system, secretion engineering, glycosylation pathway engineering, and systems metabolic engineering

Advances in synthetic biology and systems biology have led to the development of molecular techniques and modification strategies for efficient protein synthesis in *S. cerevisiae* [25]. This article systematically summarizes the engineering strategies used to enhance protein production by *S. cerevisiae*. This review examines the benefits of *S. cerevisiae* as a host for protein synthesis and categorizes its main heterologous protein products. The strategies for constructing efficient protein-producing yeast strains are summarized and discussed, including the construction of protein hyperexpression systems, protein secretion engineering, glycosylation pathway engineering, and systems metabolic engineering (Fig. 2). Moreover, potential strategies for accessing high-yield proteins and ensuring their sustainable production by *S. cerevisiae* are also proposed.

## Advantages of producing heterologous proteins by *S. cerevisiae*

*S. cerevisiae* has many advantages for protein production. First, as a domesticated microorganism with a robust history of safety, it has been frequently employed to create a wide range of recombinant proteins. The recombinant protein products have been authorized by the Food and Drug Administration (FDA) and the European Medicines Agency (EMEA) [26], and the requirement for viral detection has even been eliminated for medicinal products based on risk assessment and procedure validation [3]. In addition, *S. cerevisiae* possesses sophisticated eukaryotic structures that enable appropriate protein folding and post-translational modifications (PTMs), including acylation, glycosylation, disulfide bond formation, and hydrolysis of signal peptides, during protein production [27]. In addition, it can release proteins into the extracellular environment, facilitating further purification. Additionally, the high degree of mannose-type N-glycosylation in *S. cerevisiae* can be reduced by genetic engineering, which results in recombinant proteins with humanized glycosylation patterns, such as active antibodies [6]. Based on these biological properties, *S. cerevisiae* is a potential host for heterologous protein production.

Moreover, *S. cerevisiae* can express heterologous proteins as up to 49.3% (w/w) of its own protein [5]. *S. cerevisiae* has been commonly documented to produce heterologous proteins, and its current status is summarized in Table 1. It has been widely used for producing medicinal proteins, food proteins, and industrial enzymes. For example, several medicinal proteins, such as monoclonal antibodies, hormones, and growth factors produced by *S. cerevisiae*, are already on the market [28]. *S. cerevisiae* is also a popular choice in eukaryotic membrane proteins biosynthesis because its translate and post-translation processing are rapid, easy, and inexpensive [29]. In the foreseeable future, microbial proteins will maintain their position as a prominent modality in industry, food, and medicine. However, wild *S. cerevisiae* still suffers from protein yields well below the theoretical values, inefficient secretory transport, and other issues. Current engineering strategies for increasing *S. cerevisiae* protein output include hyperexpression systems, protein secretion engineering, glycosylation pathway engineering, and systems metabolic engineering.

**Table 1:** Examples of recombinant proteins produced in *S. cerevisiae*

Type	Protein	Titer or activity	Production scale	Reference
Medicinal Proteins	Antithrombin III	312 mg/L	Fed-batch fermentation in 5 L bioreactor	[30]
	Human pancreatic ribonuclease	0.1–0.2 mg/L	Batch fermentation in shake flask	[31]
	Transferrin	2.33 g/L	Fed-batch fermentation in 10 L bioreactor	[32]
	α-tropomyosin	20 mg/L	Batch fermentation in shake flask	[33]
	Human caseinomacropeptide	2.5 g/L	Fed-batch fermentation in 5 L bioreactor	[34]
	Human haemoglobin	18% of the total cell protein	Batch fermentations in 1 L bioreactors	[35]
	α-Amyrin	11.97 mg/L	Batch fermentation in shake flask	[36]
Food Proteins	Brazzein	9 mg/L	Batch fermentation in shake flask	[37]
	L-(+)-ergothioneine	598 ± 18 mg/L	Fed-batch fermentation in 1 L bioreactor	[38]
	Glutathione	7.3 mg/L	Batch fermentation in shake flask	[39]
	Sweet protein	0.41 g/L	Batch fermentation in shake flask	[40]
Industrial Enzymes	Laccase_3_	1176.04 U/L	Batch fermentation in shake flask	[41]
	Lipase	11,000 U/L	Fed-batch fermentation in 5 L bioreactor	[42]
	Cellulases	0.6–2.0 g/L	Batch fermentation in shake flask	[43]
	Dextranase	58.45 U/mL	Batch fermentation in shake flask	[44]
	Glucoamylase	2425 nkat/g dry cell weight	Batch fermentation in shake flask	[45]
	Levansucrase	50 U/mL	Fed-batch fermentation in 5 L bioreactor	[46]
	Cutinase	29.7 U/mL	Fed-batch fermentation in 3 L bioreactor	[47]
	Kex_2_ protease	20 mg/L	Batch fermentation in shake flask	[48]
	Collagenases	68 U/mL	Batch fermentation in shake flask	[49]
	Inulinase	34.6 U/mL	Batch fermentation in shake flask	[50]
	Cellobiohydrolase	0.58 U/g DCW	Batch fermentation in shake flask	[51]

## Construction of protein hyperexpression systems

**Fig. 3:**
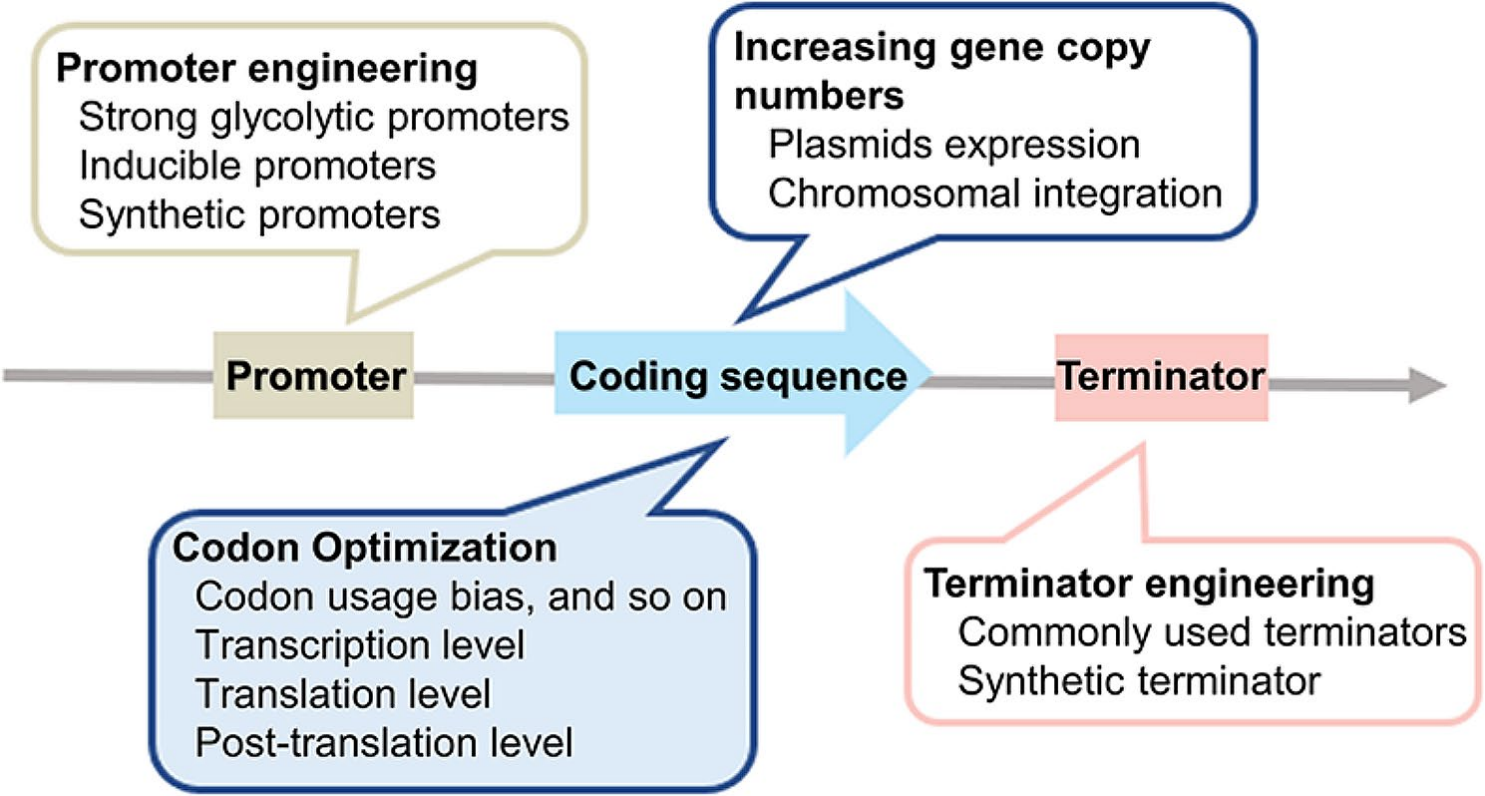
Strategies for protein hyperexpression systems, including codon optimization, increasing gene copy numbers, and transcriptional regulation

Exogenous gene expression at a high level is a key step for protein production. Certain strategies have been developed to achieve protein hyperexpression in *S. cerevisiae*. For instance, codon optimization, increasing gene copy numbers, and transcriptional regulation (including promoter and terminator engineering) have been employed to increase protein expression levels (Fig. 3).

### Codon optimization

Codon usage bias in organisms is mostly the result of genetic drift, mutation, and natural selection [52]. The efficiency of translation can be impacted by the high occurrence of rare codons. Therefore, codon-optimized gene expression is a common method for overproducing proteins and is described as “the in silico design of an optimal coding sequence for a given protein using a unique arrangement of alternative codons” [53]. The most common codon optimization strategy for matching host-specific codon use bias is to replace uncommon codons with more commonly occurring codons [54]. In addition, codon optimizations involve modifying the GC content, avoiding base repeats, eliminating restriction enzyme recognition sites, removing Chi-site extended recombination hotspots and SD ribosome binding site sequences, balancing CpG content (affecting transcription initiation), and other factors [55]. This strategy has significantly enhanced the yield of heterologous proteins in *S. cerevisiae* [45, 53, 54, 56]. Examples of these methods include the codon-optimized *T. emersonii* α-amylase variation (temA-Opt), which results in 1.6-fold extracellular activity more than natural temA, and the codon-optimized *Talaromyces emersonii* glucoamylase variation (temG-Opt), which results in 3.3-fold more extracellular activity than natural temG [53].

However, conventional codon optimization does not always improve protein expression. For example, the yields of both α-amylase and glycosylase were not improved with a codon optimization strategy [57]. Codon optimization techniques cannot guarantee optimum gene expression. There is certainly more information “hidden” in synonymous codons that is needed for protein synthesis and structure folding. Recent research has demonstrated that even changing a single codon has an impact on many processes, including the speed and accuracy of translation, the folding of proteins during cotranslation, and the protein secretion pathway [58–60]. In addition, translation efficiency can be affected by interactions among nearby codons and unsteady base pairing. To control ribosome speed and aid in protein folding, particular codons and combinations of codons are also thought to be essential [61]. Therefore, the effect of codon optimization on translational and post-translational levels should also be considered in subsequent protein production by *S. cerevisiae* [55].

### Increased gene copy number

The frequently used protein expression plasmids in *S. cerevisiae* include integration plasmids (YIp), centromeric plasmids (YCp), and episomal plasmids (YEp) [62]. YIp is stable when integrated into the yeast chromosome due to the absence of a yeast replication initiation site, although only one copy of the target gene can be added. In addition, YCp has a yeast centromere (CEN) and an autonomous replication sequence (ARS) and has high mitotic stability and a low copy number. YEp contains a 2 μm plasmid replication source and distribution site (STB or REP3) with a high copy number but low stability [63]. The copy number is the essential factor for ensuring the required level of transcription. YEp is commonly used to obtain a high copy number, which can be maintained at 10–40 copies [64]. For instance, the yields of recombinant human albumin and albumin fusion proteins can reach 5 g/L in *S. cerevisiae* with 2µ-based vector expression systems [65]. In addition, through decreasing the expression of particular marker genes and reducing the stability of marker proteins, the plasmid copy number may be increased. Chen discovered that combining the ubiquitin/N-degron tag (ubi-tag) and promoter modification of a marker gene may result in more than triple the number of 2µ-based plasmid copies [65]. However, the genetic instability of plasmids, including separation instability and structural instability, has a substantial impact on the target product yield, particularly during lengthy and intensive industrial fermentation processes [66].

In addition, chromosomal integration expression is more stable than that of free plasmids [67]. However, the amount of expression is reduced by the low copy number produced by chromosomal incorporation, necessitating an increase in the number of copies. Human alpha-fetoprotein was successfully secreted into culture medium by *S. cerevisiae* when 5–7 copies of its gene were incorporated into chromosomes [68]. In addition, the expression of heterologous genes is impacted by epigenetic changes connected to chromosomal integration sites, such as altering gene expression as a result of regulatory element interference or gene stoppage after integration into the genome’s protein-coding region [67]. It is preferable to modify the genetic structure without compromising yeast growth [66]. For example, the endo-1,4-β-glucanase ENG1 from *Aspergillus niger* was efficiently and stably secreted by integrating its gene into the HO site of *S. cerevisiae* chromosome, whose deletion did not affect yeast growth [69]. Moreover, a more practical approach is to incorporate the recombinant protein-encoding gene constructs into the noncoding genomic region of yeast. The main multicopy sites commonly used for heterologous gene integration in *S. cerevisiae* are ribosomal DNA (rDNA) and the δ site. The nontranscribed spacer (NTS) of rDNA-based cassettes was utilized to create yeast strains that produce the capsid protein of red-spotted grouper necrosis virus (RG-NNVCP) in a copy number-dependent manner. Oral treatment with altered *S. cerevisiae* containing 30 copies of the integrated RG-NNVCP cassette elicited effective immunological responses in mice [70]. In addition, the highest level of β-galactosidase, with approximately 8 gene copies, was achieved when integrated into the δ sequence of the retrotransposon Ty1 in *S. cerevisiae* [71].

### Transcriptional regulation

#### Promoter engineering

The transcription units (TUs) of a gene circuit include three biological elements: the promoter, the coding sequence (CDS), and the terminator. The functions of TUs are represented by substances encoded in CDSs, and the initiation and regulation of CDSs occur at the promoter level. Therefore, the promoter should be carefully designed and selected to ensure circuit operation as intended for synthesizing proteins [72]. The promoters most commonly employed in *S. cerevisiae* are strong glycolytic promoters and conditionally inducible promoters. Strong glycolytic promoters, such as *pTDH3*, *pPGK1*, *pTPI1*, and *pADH1*, have high levels of transcription. Conditionally inducible promoters, such as *pGAL1*, *pGAL7*, *pGAL10*, *pPHO5*, and *pMET25*, are also suitable for regulated protein expression [6]. Heterologous protein synthesis is usually expected in the late stages of *S. cerevisiae* fermentation because it can help prevent the unintentional selection of cells that grow more quickly and do not produce proteins or the production of damaging proteins [73]. The *pMET25* promoter has been utilized in *S. cerevisiae* to generate high amounts of human serum albumin (HSA), human interleukin-2, human growth hormone, HSA-fused human glucagon, and human interferon-R in medium lacking methionine [73]. Furthermore, the nitrogen catabolite repressible *GAP1* promoter has been employed to provide a high level of recombinant protein and allow for substantial biomass production in *S. cerevisiae*. This promoter has been used in yeast to produce both human membrane and soluble proteins [74].

**Fig. 4:**
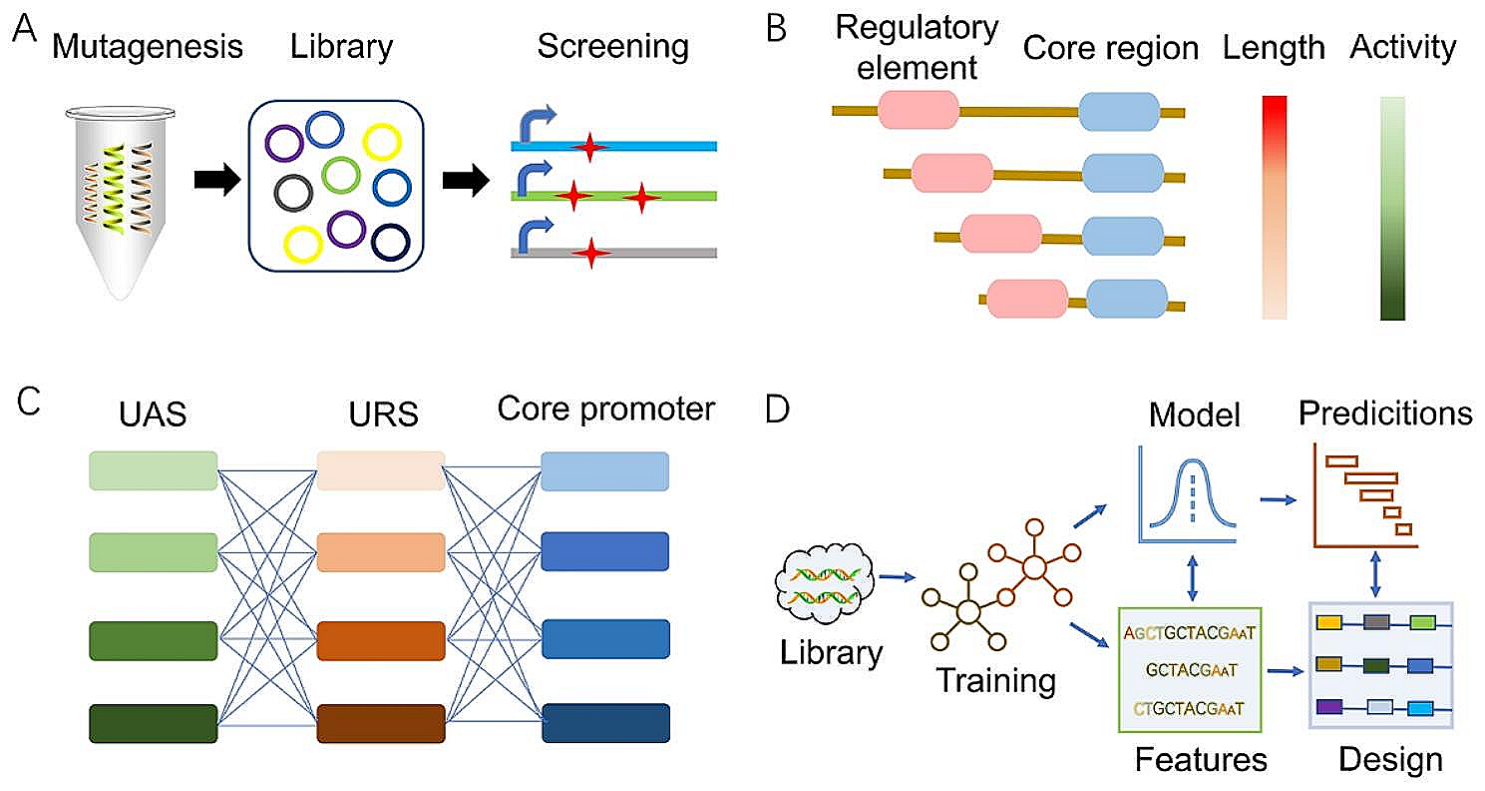
Promoter engineering for protein production in *S. cerevisiae*. (**A**) Random mutation and screening of promoter libraries. (**B**) Construction of the minimal promoter construct. (**C**) Combination of each element for hybrid promoters. (**D**) Machine learning procedures for promoter design

However, the lesser availability, poor dynamic range, and insufficient orthogonality of natural promoters further limit their applications. Therefore, promoter engineering has been proposed for designing synthetic promoters with improved properties [75]. Synthetic promoters are mainly developed by altering the sequence of natural yeast promoters through random mutagenesis, minimization, and hybridization [75] (Fig. 4A-C). In addition, the total transcript level of intron-containing genes was much greater than that of non-intron-containing genes in *S. cerevisiae* [76]. *S. cerevisiae* introns act as regulators with a 100-fold expression range, broadening the toolbox for synthetic gene expression systems and offering a foundation for accurate and stable gene expression regulation [77]. Cui et al. [78] systematically investigated protein expression by fusing introns and promoters in *S. cerevisiae* and successfully expanded the dynamic range of promoter subsets. A model for predicting the strength of intron–promoter binding was further trained to improve protein production [78]. Based on the above methods, promoter engineering can be used to obtain a broader range of gene expression to facilitate protein production.

Moreover, designing promoters with customized strengths remains challenging because (i) the mutagenesis library is dependent on the transformation efficiency of the strain and (ii) selecting from a library is difficult at high throughput [75]. As a result, several forecast models have been developed to simplify promoter design and fine-tuning. Models can be used to effectively predict protein production from promoter sequences, allowing for the quick development of relevant promoters to aid in synthetic biology studies in this model organism (Fig. 4D). Kotopka et al. [11] tested more than 327,000 sequences in an inducible promoter collection and more than 675,000 sequences in a constitutive promoter pool for gene expression activity. Subsequently, an ensemble of convolutional neural networks was trained using the aforementioned two datasets, resulting in robust predictive accuracy (R^2^ > 0.79) across various sequence-activity prediction tasks. The model-guided design approach led to extensive collections of promoters exhibiting significant sequence diversity, demonstrating greater activity than that in the training data [11]. In addition, understanding the connection between promoter sequences and expression phenotypes can help predict promoter expression strength. To create deep neural network models that are universally applicable and have great prediction performance, Vaishnav et al. [79] assessed the expression levels of millions of randomly selected promoter sequences in *S. cerevisiae*. Apart from providing a basic framework for creating regulatory sequences and providing answers to fundamental concerns concerning regulatory evolution, a method of identifying expression selection characteristics from naturally occurring variation has also been suggested [79]. The rational design of these promoters offers additional tools for expressing heterologous proteins.

#### Terminator engineering

Eukaryotic terminators play a significant role in controlling the transcription process by affecting the stability, effectiveness, and localization of mRNAs [80]. There are only a few native terminators that are commonly employed in *S. cerevisiae*, such as *CYC1t*, *TDH3t*, and *PGK1t* [81]. Green fluorescence was used as an indicator of terminator activity to quantify the activities of 5302 terminators produced from nearly 90% of the genes in *S. cerevisiae*. The activity of the top five terminators was approximately 2.5-fold greater than that of *PGK1t*, while the activity of the weakest terminator *GIC1t* was only 0.04-fold that of *PGK1t*. The wide range of gene expression regulation suggested that terminators are important elements for protein expression. In comparison to native terminators, synthetic terminators have a number of advantages, including the ability to synthesize short sequences, a low degree of sequence homology, and equal or greater functional properties. Curran et al. [82] presented a set of synthetic terminators with short (35–70 bp) lengths that may be utilized to modulate gene expression in yeast. Compared to the native *CYC1t* terminator, the best of these synthetic terminators resulted in a 3.7-fold increase in protein production and a 4.4-fold increase in transcript levels [82]. Furthermore, the expression of EGFP with a short synthetic terminator (33–66 bp) was also increased by 5.57-fold compared with that of the native *CYC1t* terminator [83]. Combining strong terminators with weak promoters can yield similar results for strong promoters. Curran et al. [84] characterized more than 30 terminators in *S. cerevisiae* and indicated that a change in the mRNA half-life is the major cause of the variation in protein and transcript expression levels. They demonstrated that when coupled with a low-expression promoter, the relative difference in output between terminators is magnified, with a maximum difference of 35-fold compared to a construct lacking a terminator and a maximum difference of 11-fold between an expression-enhancing terminator and the parent plasmid terminator [84]. Therefore, terminator engineering will be an important strategy for heterologous protein production in the future [83].

## Protein secretion engineering

**Fig. 5:**
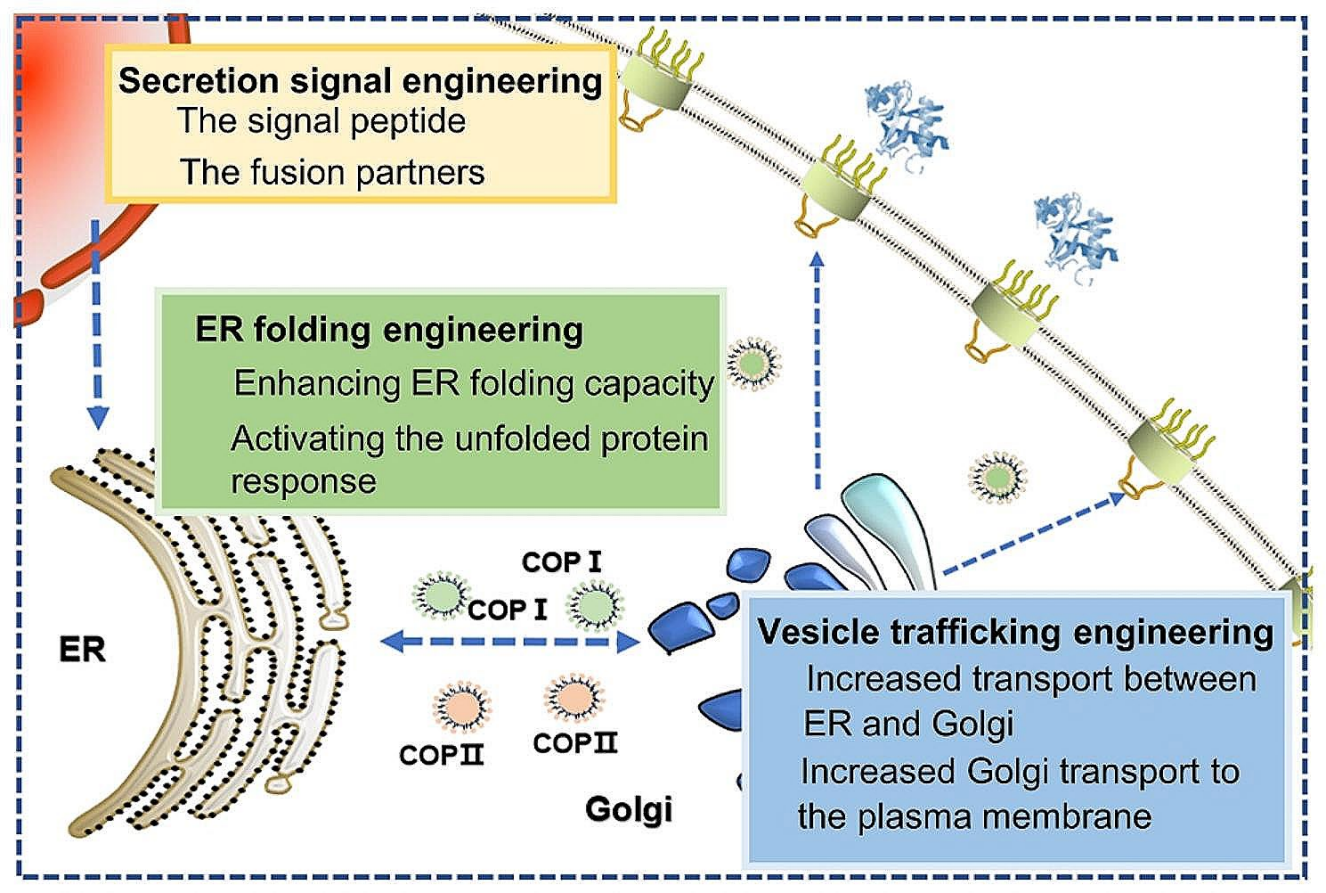
Protein secretion engineering in *S. cerevisiae*, including secretion signal engineering, ER folding engineering, and vesicle trafficking engineering

The main step in protein secretion is protein transport from endoplasmic reticulum (ER) to Golgi and further transport to the extracellular space. Increasing protein secretion can significantly enhance protein production. Moreover, the secretory system of recombinant proteins in *S. cerevisiae* is beneficial for downstream purification and large-scale industrial production, avoiding costly cell rupture, denaturation, and repeatability [66]. However, its intrinsic secretory system has certain limitations, such as hyperglycosylation, incorrect folding, inefficient secretion, and abnormal proteolytic protein processing [66]. Many recombinant proteins in *S. cerevisiae* were produced only 1% or even less of their theoretical yield, which means that they cannot reach their full potential [85]. Therefore, protein yield and quality can be significantly improved by designing and engineering protein secretion pathways. Secretion signal engineering, ER folding engineering [86], and vesicle trafficking engineering are the major strategies used to modify *S. cerevisiae* protein secretion pathway system [87] (Fig. 5).

### Secretion signal engineering

The early stages of the secretory process are influenced by the protein transport mechanism to the ER. One of the most effective approaches for promoting recombinant protein secretion is the use of secretory signal peptides [66]. The signal peptide sequences determine the secretory pathway of proteins, whether cotranslational translocation or post-translational translocation occurs in the ER, and whether the trans-Golgi network is involved [88]. However, even for recombinant proteins with minor sequence or structural differences, the secretory efficiency may vary significantly depending on the specific signal peptide sequence. It has been proven that native, exogenous, and synthetic signal peptides can guide protein secretion in *S. cerevisiae* [88]. The directed evolution of signal peptides was successfully employed to improve protein secretion. A mutant α-factor signal peptide, α_9H2_ leader, was attached to laccase, and its production was increased two-fold in *S. cerevisiae* [89]. Besides, the directed evolution of the signal peptide (MFa1pp) combined with strain engineering increased human IgG1 secretion by 180-fold [90]. Additionally, a modified version of α-factor (α_OPT_ leader) was created by using a dual (top-down and bottom-up) design strategy to optimize signal peptides. This modified form of α-factor can significantly increase the secretion of ascomycete hydrolases (a sterol esterase and two β-glucosidases) and basidiomycete oxidoreductases (aryl-alcohol oxidase, two additional laccases, and versatile peroxidase). Generally, using α_OPT_ leader increased enzyme expression levels from 2- to 20-fold higher than using α_nat_ [91]. Compared to that of the wild-type α-factor, the secretion of the insulin precursor was increased by 2.5-fold with synthetic signal peptides generated through an iterative process of rational design and empirical optimization [92].

Along with the signal peptide, the fusion partner is another powerful tool for promoting protein secretion because it increases the solubility of fusion proteins in the ER and facilitates their transport to the Golgi [93]. Protein secretion has been improved by the use of several fusion partners. These partners mainly include the cell wall proteins Scw4p and Pir4p, the cellulose-binding domain (CBD), the mitochondrial inner membrane protein UTH1, and the ER protein Voa1p [94]. Scw4p has been designed as a universal fusion partner for heterologous protein secretion in *S. cerevisiae*. Three target proteins (hGH, exendin-4, and hPTH) were fused with the C-terminally shortened Scw4p to boost their secretion, notably yielding approximately 5 g/L of exendin-4 fusion protein [93]. Lipase was effectively released with close to 90% efficiency by employing the cell wall protein Pir4 as a fusion partner, which results in approximately 400 IU of lipase activity per millilitre of cell supernatant [95]. Furthermore, a CBD from *Trichoderma harzianum* endoglucanase II (THEG) was used to promote the synthesis of *Bacillus stearothermophilus* L1 lipase, and the secretion of CBD-linker-L1 lipase increased by 7-fold, reaching approximately 1.3 g/L [96]. A N-terminal 98-amino acid domain of the mitochondrial inner membrane protein UTH1 was also employed to secrete *Rahnella aquatilis* levansucrase (RaLsrA) into the culture medium with a 63% secretion efficiency [46]. Besides, the C-terminally shortened Voa1p, an ER protein that participates in the construction of V0 sector of V-ATPase, was further developed to release small proteins, the amount of human parathyroid hormone produced was multiplied by 5-fold [94].

### ER folding engineering

Protein folding is the subsequent stage in the secretory process after translocation to the ER. The ability of the ER to fold proteins is one of the key factors restricting the secretion of recombinant proteins [66]. The unfolded protein response (UPR) is further activated by incorrectly folded peptides or an overabundance of secretory proteins which can result in luminal burden and ER stress. Multiple protective cellular processes can be induced by the UPR, such as ER-associated degradation (ERAD) of misfolded proteins and regulating protein folding [66]. Several approaches of manipulating the ER environment, including enhancing the ER folding capacity and activating the UPR, have been employed to improve protein folding ability in *S. cerevisiae* [97].

Protein folding in the ER is commonly thought to be a regulatory step in the secretion process. The overexpression of numerous folding chaperones is a basic technique. The yields of human erythropoietin and bovine prochymosin were increased by 5-fold and 26-fold, respectively, when the ATPase Hsp70 family member chaperone BiP was used to induce protein secretion in *S. cerevisiae* [98]. The collaboration of folding partners creates a diverse set of interactive networks. For example, by binding exposed hydrophobic sequences, Kar2p serves as a folding chaperone and an ER cleaner throughout the ER-associated degradation (ERAD) process. Pdi1P can catalyse the synthesis and isomerization of disulfide bonds and participate in the folding or degradation of non-disulfide proteins. The coexpression of ER chaperone Kar2p and disulfide isomerase Pdi1p could synergistically enhance the secretion of β-glucosidase by 3-fold [99].

The UPR is a widespread, coordinated reaction that eliminates misfolded proteins and enhances the oxidative environment in the ER, increasing the capacity for protein secretion. Hac1p is the main transcription factor regulating this pathway, and its overexpression can activate the entire UPR pathway and boost ER chaperone expression, thus enhancing the efficacy of heterologous protein secretion. For example, the secretion of α-amylase was increased by 2.4-fold by overexpressing *Trichoderma reesei*-derived *HAC1* in *S. cerevisiae*. The overexpression of native *S. cerevisiae HAC1* also enhanced the secretion of endogenous invertase (2-fold) and recombinant α-amylase (0.7-fold) [100]. In addition, Ire1p, a transmembrane protein that controls Hac1p synthesis by regulating mRNA splicing, is also a key component of the UPR pathway. It is essential for ER stress perception and response. Recently, the overexpression of Ire1p in mutant *S. cerevisiae* increased hepatitis B small antigen (HBsAg) production by 2.12-fold compared to that in the wild-type strain [101]. Moreover, expanding the ER appears to be a sensible course of action to prevent the detrimental consequences of protein overexpression stress and the related production of an unsaturated protein response. The deletion of the lipid-regulating gene *OPI1* resulted in an expansion of the ER in *S. cerevisiae* as well as a 4-fold increase in full-length antibody production [102].

### Vesicle trafficking engineering

The protein secretory routes involve vesicle-mediated transport processes such as protein trafficking through the ER, Golgi, trans-Golgi network, endosome, and cell membrane. The target membrane and the membrane of transport vesicles fuse at each phase of trafficking, allowing the delivery of cargo proteins [103]. The secretion of heterologous proteins has been successfully enhanced via vesicle trafficking engineering. Coat protein complex II (COPII)-encapsulated vesicles transport recombinant proteins from the ER to the Golgi. The expression of the peripheral protein Sec16 in the ER can increase ER-Golgi flux and cause additional ER membrane proteins to be directed to Golgi anterograde vesicles, resulting in a decrease in the number of ER membranes [104]. For instance, the secretions of human insulin precursor and α-amylase were increased by 34% and 16%, respectively, after Sec16 overexpression [103]. Additionally, these fundamental components are transported from the Golgi to the ER for continual anterograde transport by vesicles encased by coat protein complex I (COPI). In a background strain expressing Sec16, the GTP-activating proteins (GAPs) Gcs1 and Glo3 promoted retrograde transport from the Golgi to the ER. The increased protein secretion was resulted from the recovery and reintroduction of these components into the ER [104]. These findings suggested that the secretory route depends on a proper balance between anterograde and retrograde transport. Another secretory engineering strategy for enhancing heterologous protein synthesis in *S. cerevisiae* is to improve protein transport from the Golgi to the plasma membrane [103]. For instance, Sso1 or Sso2 is a yeast synaptic fusion protein that is involved in the fusion of Golgi-derived vesicles with the plasma membrane. The overexpression of Sso1 and Sso2 resulted in 4-fold higher α-amylase secretion [105]. Additionally, lowering intracellular retention can boost protein synthesis. Huang et al. [106] decreased intracellular heterologous protein retention and boosted the protein production capacity of yeast by 5-fold through combinatorically altering known gene targets that are involved in the secretory and trafficking pathways, as well as the histone deacetylase complex. The altered *S. cerevisiae* could produce 2.5 g/L fungal α-amylase with less than 10% of the recombinant protein was retained within the cells. Several studies have proved that selecting damaged VPS mutants in vacuoles can be used to produce various recombinant proteins more effectively, indicating that preventing errors in positioning into vacuoles can improve secretion [107]. The deletion of *VPS4*, *VPS8*, *VPS13*, *VPS35*, or *VPS36* that encode vacuolar proteases, resulted in increased production of insulin-containing fusion proteins [108]. Similarly, the deletion of *VPS10* (sorting receptor coding solution bubble hydrolase) and *PEP4* (coding solution vacuolar protease A) reduced the targeting of haemoglobin to vacuoles and protein degradation. When combined with other gene mutations, haemoglobin production was increased, and accounted for approximately 18% of its total protein content [109]. In addition, some proteins may undergo proteasome-based protein degradation [110]. For example, the deletion of extracellular protease Ski5p increased the secretion of killing toxin by approximately 10-fold [111]. The Yap3p protease as an important factor in the degradation of secreted heterologous proteins, its disruption generated a significant increase in products quality including recombinant human albumin (rHA) and rHA-human growth hormone fusion protein (rHA-hGH) [112].

## Glycosylation pathway engineering

Glycosylation occurs mainly in the ER and Golgi, and can affect protein activity, stability, and secretion [113]. Introducing or eliminating glycosylation sites at specific locations has become an important strategy for improving the production or catalytic performance of recombinant proteins [114]. Aza et al. [89] introduced N-glycosylation into laccase derived from *Pleurotus eryngii*, which improved its expression and activity in *S. cerevisiae* [89]. Glycosylation sites can also be added to increase protein secretion [115]. For instance, the secretion of keratinase was increased 5- and 1.8-fold by introducing glycosylation sites in the N-terminal and C-terminal regions, respectively [116]. Additionally, controlling N-glycan production and trimming might boost protein output. For example, the overexpression of glucosidase CWH41, which is crucial for the precise regulation of protein folding, led to a 40% increase in the amylase titre [117].

Although *S. cerevisiae* can modify proteins through glycosylation, its degree is relatively high, which can impact protein activity or lead to high allergenicity, especially for humanized proteins [114]. The extension of α-1,6-mannose can lead to hypermannosylation in *S. cerevisiae* [89]. Therefore, inhibiting the addition of the first mannose to initiate the outer chain is considered a key step in preventing hypermannosylation in *S. cerevisiae*. *OCH1* is a key gene responsible for the initial transfer of α-1,6-mannose to the outer chain [118]. Tang et al. [119] demonstrated that the deletion of *OCH1* significantly enhanced the secretion of β-glucosidase, endoglucanase, and cellobiohydrolase. The elimination of mannosylphosphates from glycans is also important for the production of humanized proteins in *S. cerevisiae*. The *MNN1, MNN4* and *MNN14* genes have been identified as being involved in mannosyl phosphorylation [120]. Kim’s study showed that in the *S. cerevisiae OCH1*Δ *MNN1*Δ *MNN4*Δ *MNN14*Δ strain, all mannose phosphorylation was abolished, which can be used to produce humanized proteins [121]. In addition, *S. cerevisiae* can also perform sugar engineering by disrupting genes encoding specific mannosyltransferases, such as *ALG3* and *OCH1* [122]. However, *ALG3* and *OCH1* mutations cause underoccupancy of N-glycosylation sites. The overexpression of *RHO1*, which encodes the Rho1p small GTPase, was confirmed to partially reverse a growth defect in *S. cerevisiae*. Therefore, *RHO1* can be used for the production of humanized proteins [123].

## Systems metabolic engineering

**Fig. 6:**
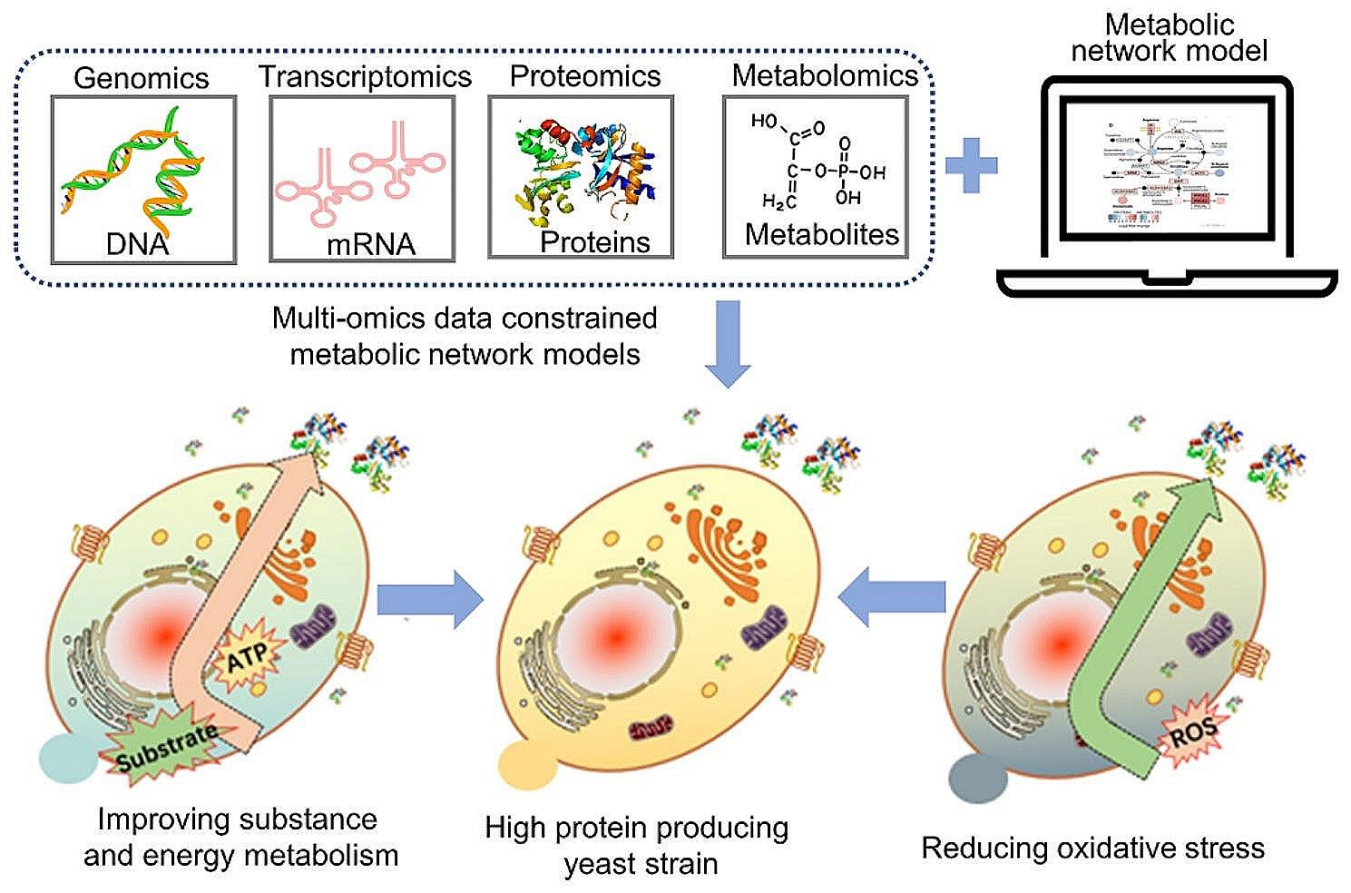
The construction of a high-protein-producing yeast assisted by systems metabolic engineering, including improving substance and energy metabolism for protein synthesis, reducing oxidative stress, and rationally engineering metabolic pathways guided by multiomics data and constrained metabolic network models

Reprogramming cellular activity is essential for reducing metabolic burden and ensuring recombinant protein production (Fig. 6) [124–126]. In addition to regulating protein expression and transport systems, efficient protein production also requires corresponding energy and precursors, which requires the analysis and engineering of metabolic pathways. The human interferon-α2a protein concentration was elevated to 276 mg/L when the key precursor adenine was uniformly introduced into the basal medium at a rate of 2 µg/mL in medium/h for 10–20 h of fermentation [127]. Additionally, Payne et al. [128] identified a *S. cerevisiae* mutant with high recombinant rHA production, which presented high gene expression levels of *LHS1*, *SCJ1*, *SIL1*, and *JEM1*, which are involved in regulating the ATPase cycle of the ER chaperone Kar2p. When these target genes were overexpressed individually or jointly, *S. cerevisiae* displayed clear advantages in the production of granulocyte–macrophage colony-stimulating factor, recombinant human transferrin, and rHA [128].

Furthermore, the overexpression of heterologous genes may result in a redox imbalance. For example, some available carbohydrates may be diverted from intended protein synthesis to unwanted byproducts [129]. As a result, growth kinetic parameters, including biomass yield, growth rate, and a particular substrate consumption rate, may be significantly influenced. Because it does not secrete numerous endogenous proteins and purifying the secreted target product is simple, *S. cerevisiae* is a desirable workhorse for the manufacture of recombinant proteins. High rates of recombinant protein synthesis place tremendous metabolic burden on yeast cells, which leads to oxidative stress and ultimately reduces their ability to produce protein. Increasing the metabolic rate of *S. cerevisiae* by overexpressing the endogenous transcription factor *HAP1* can reduce the detrimental effects of reactive oxygen species buildup associated with protein folding and consequently boost protein output [130]. Additionally, the creation of misfolded proteins or protein aggregates, which can trigger cellular oxidative stress reactions and hence restrict large-scale production, is frequently a barrier to the high-level synthesis of recombinant proteins in industrial microbes. Therefore, reducing oxidative stress can boost recombinant protein synthesis. The yield of α-amylase was successfully increased by 18.7-fold through reducing oxidative stress through enhancing membrane lipid biosynthesis, and inhibiting methionine and arginine biosynthesis [131].

Moreover, the metabolic burden is related to the additional energy cost associated with recombinant protein synthesis or the constrained transcriptional and translational resources needed to compete for the ability to produce and secrete proteins [129]. Metabolic burden regulation is a cell-wide endeavour, and modifying a single pathway has limited effects. Therefore, a metabolic network model combined with large-scale datasets (omics) has been employed to guide and regulate restriction nodes for improving protein production. For example, Ishchuk et al. [132] identified 84 genetic targets for regulating biomass and enhancing haemoglobin production based on the genome-scale metabolic model (GEM) Yeast8 of *S. cerevisiae*. In the trials, 76 genes were individually deleted or overexpressed, and 40 of these genes could enhance haemoglobin synthesis. The enzyme-constrained Yeast8 model (ecYeast8) was subsequently utilized to improve the model simulations and assess the combinatorial impact of the gene targets. Compared to the control strain, the engineered *S. cerevisiae* with 11 genetic changes generated 70-fold more intracellular haemoglobin [132]. The proteome-constrained genome-scale protein secretory model of *S. cerevisiae* (pcSecYeast) was generated by Li et al., allowing them to mimic and explain the phenotypes caused by a restricted capacity for secretion. This approach was also used to predict the targets of the overexpression of numerous recombinant proteins. Many predicted targets for high α-amylase production were validated, demonstrating the application of pcSecYeast as a computational tool to guide the efficient production of recombinant proteins [133]. Furthermore, the combination of high-throughput and omics techniques can further explain the relationship between high protein production and cellular metabolism processes. For example, Wang et al. [134] used RNAi combined with high-throughput microfluidic single-cell screening to obtain strains with improved protein secretion. The results showed that recombinant protein production can be impacted by genes involved in cell metabolism (*YDC1*, *AAD4*, *ADE8*, and *SDH1*), protein modification and degradation (*VPS73*, *KTR2*, *CNL1*, and *SSA1*), and the cell cycle (*CDC39*). Huang et al. [135] used RNA-seq to study the whole-genome transcription response of mutant yeast strains to protein secretion. The results indicated that the changes in energy metabolism could cause a decrease in respiration and an increase in fermentation, as well as a change in the balance between amino acid biosynthesis and thiamine biosynthesis. Huang et al. [136] also utilized high-throughput microfluidics to screen yeast libraries produced by UV mutagenesis. Microfluidic screening combined with whole-genome sequencing was further used to map mutations associated with increased protein secretion, identifying new engineering targets and promoting the design of new cell factories.

The invention and progression of artificial intelligence technologies have significantly enhanced the capacity for the rational construction of gene expression elements and metabolic pathways, providing valuable tools for improving protein production. The N-terminal coding sequence (NCS) is a rate-limiting step in translation and an important element in gene regulation. Wang et al. [137] applied a multiview learning strategy for NCS synthesis in *S. cerevisiae*. Two models were developed through model training and used to upregulate and downregulate gene expression. Synthetic NCS has greater than 65% accuracy, and its application has proven effective in upregulating the expression of protein-coding genes. Despite the lack of a comprehensive mechanistic understanding, the combination of big data and machine learning can facilitate the modelling of regulatory networks for gene expression and the cellular metabolome. Machine learning has recently been used to map enzyme expression patterns and utilize them to predict metabolite concentrations [138]. Modulations in enzyme expression can influence metabolite levels through synergistic interactions. Zelezniak et al. [139] demonstrated the feasibility of employing machine learning to chart regulatory enzyme expression patterns, and predict the metabolome of kinase-deficient cells using the enzyme expression proteome. Their research quantified the impact of enzyme abundance on metabolic regulation, unveiling the potential of machine learning for comprehending intricate metabolic regulatory processes.

## Conclusions

*S. cerevisiae* has been widely employed to produce heterologous proteins due to its biological advantages. However, the yield of proteins in wild yeast is much lower than the theoretical value. To achieve efficient biosynthesis of target proteins, the coordination between heterologous protein genes and *S. cerevisiae* chassis is particularly critical. Currently, researches on high protein production by *S. cerevisiae* are being conducted in the areas of expression systems, secretion engineering, glycosylation engineering and systems metabolic engineering. However, most *S. cerevisiae* protein products are produced at low yields, and there is still a large gap between theoretical and actual application. With the rapid progress in synthetic biology and the idea of “carbon neutral” method, *S. cerevisiae* strains with high yields of protein might be generated in the future in the following respects.

The development of novel gene editing tools has greatly improved the speed and efficiency of *S. cerevisiae* genome engineering. CRISPR-based systems have exhibited advantages in gene editing and heterologous metabolic pathway assembly, allowing simultaneous multiple gene editing without screening markers, greatly reducing the cycle time for heterologous metabolic pathway introduction and gene-targeted mutations, and enabling the optimization of individual genes or combinations of gene metabolic networks. With the development of the CRISPR system, the exploitation of Cas proteins has made this technology more promising for protein synthesis in *S. cerevisiae*. In addition, the genome design and reconfiguration of *S. cerevisiae* can enable the cell factory to obtain new functions and potentially evolve, suggesting the possibility of rapidly constructing efficient cell factories. For instance, synthetic chromosome recombination and modification by LoxP-mediated evolution (SCRaMbLE) can introduce genome rearrangement events in *S. cerevisiae*. An effective method for high protein production is made possible by this technology, which enables researchers to investigate the interactions of various rearrangements, the contribution of gene position throughout the genome, and gene copy number.

In addition, because metabolism and other biological processes are complicated, the construction of highly accurate dynamic cellular models remains a major challenge. With the development of high-throughput systems biology data, the generation of high-quality yeast experimental datasets will further promote our understanding of *S. cerevisiae* behaviour at the quantitative and dynamic levels. The combination of genomics, transcriptomics, proteomics, and metabolomics can provide comprehensive biological information to reveal the cell state under different conditions, determine important nodes limiting protein production, and allow reasonable tailoring of the gene network. Furthermore, deep learning (DL) is essential for the systematic analysis of heterologous data that cannot be discovered by histological techniques. This approach enables a better understanding of the underlying biological processes. Furthermore, an increasing number of DL-based computing strategies are being developed via specialized platforms. As a result of the development of these accurate, data-driven models, they may be used to create efficient systems for *S. cerevisiae* heterologous protein synthesis.

In terms of feedstock, *S. cerevisiae* can utilize not only sugar as the first-generation (1G) feedstock, but also industrial and agricultural waste as the second-generation (2G) feedstock, and single carbon compounds as the third-generation (3G) feedstock. The 1G feedstock comprises glucose, arabinose [140], and xylose [20, 21], among others. In addition to sugary waste, 2G feedstocks include discarded glycerol [22, 23], cellulose [141], etc. Importantly, 3G feedstocks are more abundant, less expensive, and carbon neutral, contributing to alleviating the energy crisis and reducing greenhouse gases. *S. cerevisiae* has been engineered to utilize CH_3_OH [24] for cell growth. Therefore, engineering *S. cerevisiae* to utilize 3G for protein production is an important direction that will not only help reduce protein costs but also benefit carbon-neutral targets. It is possible to engineer *S. cerevisiae* as a strong biological chassis for effective protein synthesis.

## References

[1] Arnthong J, Ponjarat J, Bussadee P, Deenarn P, Prommana P, Phienluphon A, Charoensri S, Champreda V, Zhao X-Q, Suwannarangsee S. Enhanced surface display efficiency of β-glucosidase in *Saccharomyces cerevisiae* by disruption of cell wall protein-encoding genes *YGP1* and *CWP2*. Biochem Eng J. 2022;179.

[2] Ren S, Hu P, Jia J, Ni J, Jiang T, Yang H, Bai J, Tian C, Chen L, Huang Q et al. Engineering of *Saccharomyces cerevisiae* for sensing sweetness. Biochem Eng J. 2022;177.

[3] Love KR, Dalvie NC, Love JC (2018). The yeast stands alone: the future of protein biologic production. Curr Opin Biotechnol.

[4] Arbige MV, Shetty JK, Chotani GK (2019). Industrial Enzymology: the next chapter. Trends Biotechnol.

[5] Fasolin LH, Pereira RN, Pinheiro AC, Martins JT, Andrade CCP, Ramos OL, Vicente AA (2019). Emergent food proteins-towards sustainability, health and innovation. Food Res Int.

[6] Wang G, Huang M, Nielsen J (2017). Exploring the potential of *Saccharomyces cerevisiae* for biopharmaceutical protein production. Curr Opin Biotechnol.

[7] Lei Q, Ma J, Du G, Zhou J, Guan X (2023). Efficient expression of a cytokine combination in *Saccharomyces cerevisiae* for cultured meat production. Food Res Int.

[8] Tuomisto HL (2022). Environmental benefits of eating mycoprotein. Nature.

[9] Hashempour-Baltork F, Jannat B, Dadgarnejad M, Mirza Alizadeh A, Khosravi-Darani K, Hosseini H (2023). Mycoprotein as chicken meat substitute in nugget formulation: Physicochemical and sensorial characterization. Food Sci Nutr.

[10] Gamarra-Castillo O, Echeverry-Montaña N, Marbello-Santrich A, Hernández-Carrión M, Restrepo S. Meat substitute development from fungal protein (*Aspergillus oryzae*). Foods*.* 2022;11. 10.3390/foods11192940 PMC9563988 36230014

[11] Kotopka BJ, Smolke CD (2020). Model-driven generation of artificial yeast promoters. Nat Commun.

[12] Zampieri G, Vijayakumar S, Yaneske E, Angione C (2019). Machine and deep learning meet genome-scale metabolic modeling. PLoS Comput Biol.

[13] Kim Y, Kim GB, Lee SY (2021). Machine learning applications in genome-scale metabolic modeling. Curr Opin Syst Biology.

[14] Chen Y, Li F, Nielsen J (2022). Genome-scale modeling of yeast metabolism: retrospectives and perspectives. FEMS Yeast Res.

[15] Lian J, HamediRad M, Zhao H (2018). Advancing metabolic engineering of *Saccharomyces cerevisiae* using the CRISPR/Cas system. Biotechnol J.

[16] Richardson SM, Mitchell LA, Stracquadanio G, Yang K, Dymond JS, DiCarlo JE, Lee D, Huang CLV, Chandrasegaran S, Cai Y (2017). Design of a synthetic yeast genome. Science.

[17] Zhou S, Wu Y, Xie ZX, Jia B, Yuan YJ (2021). Directed genome evolution driven by structural rearrangement techniques. Chem Soc Rev.

[18] Thak EJ, Yoo SJ, Moon HY, Kang HA. Yeast synthetic biology for designed cell factories producing secretory recombinant proteins. FEMS Yeast Res. 2020;20. 10.1093/femsyr/foaa009 32009173

[19] Tripathi NK, Shrivastava A (2019). Recent developments in bioprocessing of recombinant proteins: expression hosts and process development. Front Bioeng Biotechnol.

[20] Bettiga M, Bengtsson O, Hahn-Hagerdal B, Gorwa-Grauslund MF (2009). Arabinose and xylose fermentation by recombinant *Saccharomyces cerevisiae* expressing a fungal pentose utilization pathway. Microb Cell Fact.

[21] Wang C, Zhao J, Qiu C, Wang S, Shen Y, Du B, Ding Y, Bao X (2017). Coutilization of D-glucose, D-xylose, and L-arabinose in *Saccharomyces cerevisiae* by coexpressing the metabolic pathways and evolutionary engineering. Biomed Res Int.

[22] Nan W, Zhao F, Zhang C, Ju H, Lu W (2020). Promotion of compound K production in *Saccharomyces cerevisiae* by glycerol. Microb Cell Fact.

[23] Yu KO, Kim SW, Han SO (2010). Engineering of glycerol utilization pathway for ethanol production by *Saccharomyces cerevisiae*. Bioresour Technol.

[24] Zhan C, Li X, Lan G, Baidoo EEK, Yang Y, Liu Y, Sun Y, Wang S, Wang Y, Wang G (2023). Reprogramming methanol utilization pathways to convert *Saccharomyces cerevisiae* to a synthetic methylotroph. Nat Catal.

[25] Ting T-Y, Li Y, Bunawan H, Ramzi AB, Goh H-H (2023). Current advancements in systems and synthetic biology studies of *Saccharomyces cerevisiae*. J Biosci Bioeng.

[26] Ganeva V, Stefanova D, Angelova B, Galutzov B, Velasco I, Arévalo-Rodríguez M (2015). Electroinduced release of recombinant β-galactosidase from *Saccharomyces cerevisiae*. J Biotechnol.

[27] Liu F, Wu X, Li L, Liu Z, Wang Z (2013). Use of baculovirus expression system for generation of virus-like particles: successes and challenges. Protein Exp Purif.

[28] Nielsen J (2013). Production of biopharmaceutical proteins by yeast: advances through metabolic engineering. Bioengineered.

[29] Cartwright SP, Mikaliunaite L, Bill RM. Membrane protein production in the yeast, *S. cerevisiae*. Method Microbiol*.* 2016;1432:23–35. 10.1007/978-1-4939-3637-3_2 27485327

[30] Mallu MR, Vemula S, Ronda SR (2016). Production, purification and characterization of recombinant human antithrombin III by *Saccharomyces cerevisiae*. Electron J Biotechnol.

[31] Ribó M, delCardayré SB, Raines RT, de Llorens R, Cuchillo CM (1996). Production of human pancreatic ribonuclease in *Saccharomyces cerevisiae* and *Escherichia coli*. Protein Exp Purif.

[32] Finnis CJA, Payne T, Hay J, Dodsworth N, Wilkinson D, Morton P, Saxton MJ, Tooth DJ, Evans RW, Goldenberg H (2010). High-level production of animal-free recombinant transferrin from *Saccharomyces cerevisiae*. Microb Cell Fact.

[33] Alegria MC, Lavarda SCS, Lataro RC, Hilario E, Ferro JA, Bertolini MC (2003). Conditions affecting production of functional muscle recombinant α-tropomyosin in *Saccharomyces cerevisiae*. Protein Exp Purif.

[34] Kim Y-J, Oh Y-K, Kang W, Lee EY, Park S (2005). Production of human caseinomacropeptide in recombinant *Saccharomyces cerevisiae* and *Pichia pastoris*. J Ind Microbiol Biotechnol.

[35] Ishchuk OP, Frost AT, Muñiz-Paredes F, Matsumoto S, Laforge N, Eriksson NL, Martínez JL, Petranovic D (2021). Improved production of human hemoglobin in yeast by engineering hemoglobin degradation. Metab Eng.

[36] Yu Y, Chang P, Yu H, Ren H, Hong D, Li Z, Wang Y, Song H, Huo Y, Li C (2018). Productive amyrin synthases for efficient α-amyrin synthesis in engineered *Saccharomyces cerevisiae*. ACS Synth Biol.

[37] Kazemi-Nasab A, Shahpiri A (2020). Expression of brazzein, a small sweet-tasting protein in *Saccharomyces cerevisiae*: an introduction for production of sweet yeasts. Protein & Peptide Letters.

[38] Van der Hoek SA, Darbani B, Zugaj KE, Prabhala BK, Biron MB, Randelovic M, Medina JB, Kell DB, Borodina I. Engineering the yeast *Saccharomyces cerevisiae* for the production of l-(+)-ergothioneine. Front Bioeng Biotechnol. 2019;7. 10.3389/fbioe.2019.00262 PMC6797849 31681742

[39] Hara KY, Kim S, Yoshida H, Kiriyama K, Kondo T, Okai N, Ogino C, Fukuda H, Kondo A (2012). Development of a glutathione production process from proteinaceous biomass resources using protease-displaying *Saccharomyces cerevisiae*. Appl Microbiol Biotechnol.

[40] Chen Z, Li Z, Yu N, Yan L (2011). Expression and secretion of a single-chain sweet protein, monellin, in *Saccharomyces cerevisiae* by an alpha-factor signal peptide. Biotechnol Lett.

[41] Jeong YS, So KK, Lee JH, Kim JM, Chun GT, Chun J, Kim DH (2019). Optimization of growth medium and fermentation conditions for the production of laccase_3_ from cryphonectria parasitica using recombinant *Saccharomyces cerevisiae*. Mycobiology.

[42] Whang J, Ahn J, Chun C-S, Son Y-J, Lee H, Choi E-S (2009). Efficient, galactose-free production of Candida Antarctica lipase B by GAL10 promoter in ∆gal80 mutant of *Saccharomyces cerevisiae*. Process Biochem.

[43] Lee C-R, Sung BH, Lim K-M, Kim M-J, Sohn MJ, Bae J-H, Sohn J-H (2017). Co-fermentation using recombinant *Saccharomyces cerevisiae* yeast strains hyper-secreting different cellulases for the production of cellulosic bioethanol. Sci Rep.

[44] Liu J, Sun Q, Yin H, Wang L, Wei H, Li K, Hang F (2020). Optimal fermentation of *Saccharomyces cerevisiae* expressing a dextranase from chaetomium gracile. Sugar Tech.

[45] Favaro L, Jooste T, Basaglia M, Rose SH, Saayman M, Görgens JF, Casella S, van Zyl WH (2012). Codon-optimized glucoamylase sGAI of *aspergillus awamori* improves starch utilization in an industrial yeast. Appl Microbiol Biotechnol.

[46] Ko H, Bae J-H, Sung BH, Kim M-J, Kim C-H, Oh B-R, Sohn J-H (2019). Efficient production of levan using a recombinant yeast *Saccharomyces cerevisiae* hypersecreting a bacterial levansucrase. J Ind Microbiol Biotechnol.

[47] Zhang J, Cai Y, Du G, Chen J, Wang M, Kang Z (2017). Evaluation and application of constitutive promoters for cutinase production by *Saccharomyces cerevisiae*. J Microbiol.

[48] Kim M-J, Sung BH, Kim H-J, Sohn J-H, Bae J-H (2022). Production of autolysis-proof Kex_2_ protease from Candida albicans in *Saccharomyces cerevisiae* for in vitro processing of fusion proteins. Appl Microbiol Biotechnol.

[49] Xiao H, Liu X, Feng Y, Zheng L, Zhao M, Huang M (2022). Secretion of collagenases by *Saccharomyces cerevisiae* for collagen degradation. Biotechnol Biofuels Bioprod.

[50] Seok-Hwan, Lim H, Lee D-E. Sok, Eui-Sung, Choi: Recombinant production of an inulinase in a *Saccharomyces cerevisiae* gal80 strain. J Microbiol Biotechn*.* 2010. 10.4014/jmb.1007.07009 21124058

[51] Kroukamp H, den Haan R, la Grange DC, Sibanda N, Foulquié-Moreno MR, Thevelein JM, van Zyl WH (2017). Strain breeding enhanced heterologous cellobiohydrolase secretion by *Saccharomyces cerevisiae* in a protein specific manner. Biotechnol J.

[52] Papamichail D, Liu H, Machado V, Gould N, Coleman JR, Papamichail G (2018). Codon context optimization in synthetic gene design. IEEE/ACM Trans Comput Biol Bioinform.

[53] Cripwell RA, Rose SH, Viljoen-Bloom M, Van ZWH (2019). Improved raw starch amylase production by *Saccharomyces cerevisiae* using codon optimisation strategies. FEMS Yeast Res.

[54] Lanza AM, Curran KA, Rey LG, Alper HS (2014). A condition-specific codon optimization approach for improved heterologous gene expression in *Saccharomyces cerevisiae*. BMC Syst Biol.

[55] Yang YPMX, Huo YX (2019). Application of codon optimization strategy in heterologous protein expression. Chin J Biotechnol.

[56] Kaishima M, Ishii J, Matsuno T, Fukuda N, Kondo A (2016). Expression of varied GFPs in *Saccharomyces cerevisiae*: Codon optimization yields stronger than expected expression and fluorescence intensity. Sci Rep.

[57] Cripwell RA, Rose SH, van Zyl WH (2017). Expression and comparison of codon optimised *aspergillus tubingensis* amylase variants in *Saccharomyces cerevisiae*. FEMS Yeast Res.

[58] Shah K, Cheng Y, Hahn B, Bridges R, Bradbury NA, Mueller DM (2015). Synonymous codon usage affects the expression of wild type and F508del CFTR. J Mol Biol.

[59] Plotkin JB, Kudla G (2011). Synonymous but not the same: the causes and consequences of codon bias. Nat Rev Genet.

[60] Chevance FFVLGS, Hughes KT. The effects of codon context on in vivo translation speed. PLoS Genet. 2014;10. 10.1371/journal.pgen.1004392 PMC4046918 24901308

[61] Brule CE, Grayhack EJ (2017). Synonymous codons: choose wisely for expression. Trends Genet.

[62] Vieira Gomes AM, Souza Carmo T, Silva Carvalho L, Mendonça Bahia F, Parachin NS. Comparison of yeasts as hosts for recombinant protein production. Microorganisms*.* 2018;6. 10.3390/microorganisms6020038 PMC6027275 29710826

[63] Yang J, Tian Y, Liu H, Kan Y, Zhou Y, Wang Y, Luo Y. Harnessing the endogenous 2µ plasmid of *Saccharomyces cerevisiae* for pathway construction. Front Microbiol. 2021;12. 10.3389/fmicb.2021.679665 PMC8249740 34220765

[64] Da Silva NA, Srikrishnan S (2012). Introduction and expression of genes for metabolic engineering applications in *Saccharomyces cerevisiae*. FEMS Yeast Res.

[65] Chen Y, Partow S, Scalcinati G, Siewers V, Nielsen J (2012). Enhancing the copy number of episomal plasmids in *Saccharomyces cerevisiae* for improved protein production. FEMS Yeast Res.

[66] Thak EJ, Yoo SJ, Moon HY, Kang HA (2020). Yeast synthetic biology for designed cell factories producing secretory recombinant proteins. FEMS Yeast Res.

[67] Bai Flagfeldt D, Siewers V, Huang L, Nielsen J (2009). Characterization of chromosomal integration sites for heterologous gene expression in *Saccharomyces cerevisiae*. Yeast.

[68] Dudich E, Dudich I, Semenkova L, Benevolensky S, Morozkina E, Marchenko A, Zatcepin S, Dudich D, Soboleva G, Khromikh L (2012). Engineering of the *Saccharomyces cerevisiae* yeast strain with multiple chromosome-integrated genes of human alpha-fetoprotein and its high-yield secretory production, purification, structural and functional characterization. Protein Exp Purif.

[69] Taipakova SM, Smekenov IT, Saparbaev MK, Bissenbaev AK (2015). Characterization of *Aspergillus* Niger endo-1,4-beta-glucanase ENG1 secreted from *Saccharomyces cerevisiae* using different expression vectors. Genet Mol Res.

[70] Moon HY, Lee DW, Sim GH, Kim H-J, Hwang JY, Kwon M-G, Kang B-K, Kim JM, Kang HA (2016). A new set of rDNA-NTS-based multiple integrative cassettes for the development of antibiotic-marker-free recombinant yeasts. J Biotechnol.

[71] Oliveira C, Teixeira JA, Lima N, Da Silva NA, Domingues L (2007). Development of stable flocculent *Saccharomyces cerevisiae* strain for continuous *aspergillus Niger* β-galactosidase production. J Biosci Bioeng.

[72] Feng X, Marchisio MA. *Saccharomyces cerevisiae* promoter engineering before and during the synthetic biology era. Biology*.* 2021;10. 10.3390/biology10060504 PMC8229000 34204069

[73] Solow SP, Sengbusch J, Laird MW (2005). Heterologous protein production from the inducible MET25 promoter in *Saccharomyces cerevisiae*. Biotechnol Prog.

[74] Debailleul F, Trubbia C, Frederickx N, Lauwers E, Merhi A, Ruysschaert J-M, André B, Govaerts C (2013). Nitrogen catabolite repressible GAP1 promoter, a new tool for efficient recombinant protein production in *S. Cerevisiae*. Microb Cell Fact.

[75] Tang H, Wu Y, Deng J, Chen N, Zheng Z, Wei Y, Luo X, Keasling JD. Promoter architecture and promoter engineering in *Saccharomyces cerevisiae*. Metabolites*.* 2020;10. 10.3390/metabo10080320 PMC7466126 32781665

[76] Juneau K, Miranda M, Hillenmeyer ME, Nislow C, Davis RW (2006). Introns regulate RNA and protein abundance in yeast. Genetics.

[77] Yofe IZZ, Blau R, Schuldiner M, Tuller T (2014). Accurate, model-based tuning of synthetic gene expression using introns in *S. Cerevisiae*. PLoS Genet.

[78] Cui X, Ma X, Prather KLJ, Zhou K (2021). Controlling protein expression by using intron-aided promoters in *Saccharomyces cerevisiae*. Biochem Eng J.

[79] Vaishnav ED, de Boer CG, Molinet J, Yassour M, Fan L, Adiconis X, Thompson DA, Levin JZ, Cubillos FA, Regev A (2022). The evolution, evolvability and engineering of gene regulatory DNA. Nature.

[80] Wei L, Wang Z, Zhang G, Ye B (2017). Characterization of terminators in *Saccharomyces cerevisiae* and an exploration of factors affecting their strength. ChemBioChem.

[81] Yamanishi M, Katahira S, Matsuyama T (2011). TPS1 terminator increases mRNA and protein yield in a *Saccharomyces cerevisiae* expression system. Biosci Biotechnol Biochem.

[82] Curran KA, Morse NJ, Markham KA, Wagman AM, Gupta A, Alper HS (2015). Short synthetic terminators for improved heterologous gene expression in yeast. ACS Synth Biol.

[83] Ahmed MS, Ikram S, Rasool A, Li C (2019). Design and construction of short synthetic terminators for β-amyrin production in *Saccharomyces cerevisiae*. Biochem Eng J.

[84] Curran KA, Karim AS, Gupta A, Alper HS (2013). Use of expression-enhancing terminators in *Saccharomyces cerevisiae* to increase mRNA half-life and improve gene expression control for metabolic engineering applications. Metab Eng.

[85] Liu Z, Liu L, Österlund T, Hou J, Huang M, Fagerberg L, Petranovic D, Uhlén M, Nielsen J (2014). Improved production of a heterologous amylase in *Saccharomyces cerevisiae* by inverse metabolic engineering. Appl Environ Microbiol.

[86] Lin Y, Feng Y, Zheng L, Zhao M, Huang M (2023). Improved protein production in yeast using cell engineering with genes related to a key factor in the unfolded protein response. Metab Eng.

[87] Yang S, Shen J, Deng J, Li H, Zhao J, Tang H, Bao X. Engineering cell polarization improves protein production in *Saccharomyces cerevisiae*. Microorganisms*.* 2022;10. 10.3390/microorganisms10102005 PMC9609600 36296281

[88] Hou J, Tyo KEJ, Liu Z, Petranovic D, Nielsen J (2012). Metabolic engineering of recombinant protein secretion by *Saccharomyces cerevisiae*. FEMS Yeast Res.

[89] Aza P, de Salas F, Molpeceres G, Rodríguez-Escribano D, de la Fuente I, Camarero S. Protein engineering approaches to enhance fungal laccase production in *S. Cerevisiae*. Int J Mol Sci. 2021;22. 10.3390/ijms22031157 PMC7866195 33503813

[90] Rakestraw JA, Sazinsky SL, Piatesi A, Antipov E, Wittrup KD (2009). Directed evolution of a secretory leader for the improved expression of heterologous proteins and full-length antibodies in *Saccharomyces cerevisiae*. Biotechnol Bioeng.

[91] Aza P, Molpeceres G, de Salas F, Camarero S (2021). Design of an improved universal signal peptide based on the α-factor mating secretion signal for enzyme production in yeast. Cell Mol Life Sci.

[92] Kjeldsen T, Pettersson AF, Hach M, Diers I, Havelund S, Hansen PH, Andersen AS (1997). Synthetic leaders with potential BiP binding mediate high-yield secretion of correctly folded insulin precursors from *Saccharomyces cerevisiae*. Protein Exp Purif.

[93] Bae J-H, Yun S-H, Kim M-J, Kim H-J, Sung BH, Kim SI, Sohn J-H (2022). Secretome-based screening of fusion partners and their application in recombinant protein secretion in *Saccharomyces cerevisiae*. Appl Microbiol Biotechnol.

[94] Bae J-H, Sung BH, Seo J-W, Kim CH, Sohn J-H (2016). A novel fusion partner for enhanced secretion of recombinant proteins in *Saccharomyces cerevisiae*. Appl Microbiol Biotechnol.

[95] Mormeneo M, Andrés I, Bofill C, Díaz P, Zueco J (2008). Efficient secretion of *Bacillus subtilis* lipase A in *Saccharomyces cerevisiae* by translational fusion to the Pir4 cell wall protein. Appl Microbiol Biotechnol.

[96] Ahn JO, Choi ES, Lee HW, Hwang SH, Kim CS, Jang HW, Haam SJ, Jung JK (2004). Enhanced secretion of *Bacillus stearothermophilus* L1 lipase in *Saccharomyces cerevisiae* by translational fusion to cellulose-binding domain. Appl Microbiol Biotechnol.

[97] Tang H, Bao X, Shen Y, Song M, Wang S, Wang C, Hou J (2015). Engineering protein folding and translocation improves heterologous protein secretion in *Saccharomyces cerevisiae*. Biotechnol Bioeng.

[98] Idiris A, Tohda H, Kumagai H, Takegawa K (2010). Engineering of protein secretion in yeast: strategies and impact on protein production. Appl Microbiol Biotechnol.

[99] Smith JD, Tang BC, Robinson AS (2004). Protein disulfide isomerase, but not binding protein, overexpression enhances secretion of a non-disulfide-bonded protein in yeast. Biotechnol Bioeng.

[100] Valkonen M, Penttilä M, Saloheimo M (2003). Effects of inactivation and constitutive expression of the unfolded- protein response pathway on protein production in the yeast *Saccharomyces cerevisiae*. Appl Environ Microbiol.

[101] Sheng J, Flick H, Feng X. Systematic optimization of protein secretory pathways in *Saccharomyces cerevisiae* to increase expression of hepatitis B small antigen. Front Microbiol. 2017;8. 10.3389/fmicb.2017.00875 PMC5432677 28559891

[102] de Ruijter JC, Koskela EV, Frey AD (2016). Enhancing antibody folding and secretion by tailoring the *Saccharomyces cerevisiae* endoplasmic reticulum. Microb Cell Fact.

[103] Hou J, Tyo K, Liu Z, Petranovic D, Nielsen J (2012). Engineering of vesicle trafficking improves heterologous protein secretion in *Saccharomyces cerevisiae*. Metab Eng.

[104] Bao J, Huang M, Petranovic D, Nielsen J (2018). Balanced trafficking between the ER and the golgi apparatus increases protein secretion in yeast. AMB Express.

[105] Toikkanen JH, Sundqvist L, Keränen S (2004). *Kluyveromyces lactis SSO1* and *SEB1* genes are functional in *Saccharomyces cerevisiae* and enhance production of secreted proteins when overexpressed. Yeast.

[106] Huang M, Wang G, Qin J, Petranovic D, Nielsen J. Engineering the protein secretory pathway of *Saccharomyces cerevisiae* enables improved protein production. PNAS*.* 2018;115:11025–11032. 10.1073/pnas.1809921115 PMC6255153 30397111

[107] Em TGS. Compartmental organization of Golgi-specific protein modification and vacuolar protein sorting events defined in a yeast Sec18 (NSF) mutant. J Cell Biol. 1991;2. 10.1083/jcb.114.2.207 PMC2289075 2071670

[108] Zhang B-y, Chang A, Kjeldsen TB, Arvan P (2001). Intracellular retention of newly synthesized insulin in yeast is caused by endoproteolytic processing in the golgi complex. J Cell Biol.

[109] Liu Z, Liu L, Osterlund T, Hou J, Huang M, Fagerberg L, Petranovic D, Uhlen M, Nielsen J (2014). Improved production of a heterologous amylase in *Saccharomyces cerevisiae* by inverse metabolic engineering. Appl Environ Microbiol.

[110] Sagt CMJ, Müller WH, van der Heide L, Boonstra J, Verkleij AJ, Verrips CT (2002). Impaired cutinase secretion in *Saccharomyces cerevisiae* induces irregular endoplasmic reticulum (ER) membrane proliferation, oxidative stress, and ER-associated degradation. Appl Environ Microbiol.

[111] Howard Bussey OSaDS (1983). Protein secretion in yeast: two chromosomal mutants that oversecrete killer toxin in *Saccharomyce scerevisiae*. Curr Genet.

[112] Kerry-Williams SM, Gilbert SC, Evans LR, Ballance DJ (1998). Disruption of the *Saccharomyces cerevisiae YAP3* gene reduces the proteolytic degradation of secreted recombinant human albumin. Yeast.

[113] Ge F, Zhu L, Aang A, Song P, Li W, Tao Y, Du G (2018). Recent advances in enhanced enzyme activity, thermostability and secretion by N-glycosylation regulation in yeast. Biotechnol Lett.

[114] Hamilton SR, Gerngross TU (2007). Glycosylation engineering in yeast: the advent of fully humanized yeast. Curr Opin Biotechnol.

[115] Piirainen MA, Frey AD (2020). Investigating the role of ERAD on antibody processing in glycoengineered *Saccharomyces cerevisiae*. FEMS Yeast Res.

[116] Sagt CMJ, Kleizen B, Verwaal R, de Jong MDM, Müller WH, Smits A, Visser C, Boonstra J, Verkleij AJ, Verrips CT (2000). Introduction of an N-Glycosylation site increases secretion of heterologous proteins in yeasts. Appl Environ Microbiol.

[117] Qi Q, Li F, Yu R, Engqvist Martin KM, Siewers V, Fuchs J, Nielsen J (2020). Different routes of protein folding contribute to improved protein production in *Saccharomyces cerevisiae*. mBio.

[118] Harris SL, Waters MG (1996). Localization of a yeast early golgi mannosyltransferase, Och1p, involves retrograde transport. J Cell Biol.

[119] Tang H, Wang S, Wang J, Song M, Xu M, Zhang M, Shen Y, Hou J, Bao X (2016). N-hypermannose glycosylation disruption enhances recombinant protein production by regulating secretory pathway and cell wall integrity in *Saccharomyces cerevisiae*. Sci Rep.

[120] Gil JY, Park J-N, Lee KJ, Kang J-Y, Kim YH, Kim S, Kim S-Y, Kwon O, Lim YT, Kang HA, Oh D-B (2015). Increased mannosylphosphorylation of N-glycans by heterologous expression of YlMPO1 in glyco-engineered *Saccharomyces cerevisiae* for mannose-6-phosphate modification. J Biotechnol.

[121] Kim YH, Kang J-Y, Gil JY, Kim S-Y, Shin KK, Kang HA, Kim J-Y, Kwon O, Oh D-B (2017). Abolishment of N-glycan mannosylphosphorylation in glyco-engineered *Saccharomyces cerevisiae* by double disruption of *MNN4* and *MNN14* genes. Appl Microbiol Biotechnol.

[122] Chiba Y, Jigami Y (2007). Production of humanized glycoproteins in bacteria and yeasts. Curr Opin Chem Biol.

[123] Xu S, Zhang G-Y, Zhang H, Kitajima T, Nakanishi H, Gao X-D (2016). Effects of *Rho1*, a small GTPase on the production of recombinant glycoproteins in *Saccharomyces cerevisiae*. Microb Cell Fact.

[124] Corte L, Roscini L, Pierantoni DC, Pellegrino RM, Emiliani C, Basaglia M, Favaro L, Casella S, Cardinali G. Delta-Integration of single gene shapes the whole metabolomic short-term response to ethanol of recombinant *Saccharomyces cerevisiae* strains. Metabolites. 2020;10. 10.3390/metabo10040140 PMC7241245 32260275

[125] Shioya S, Shimizu H, Hirasawa T, Nagahisa K, Furusawa C, Pandey G, Katakura Y (2007). Metabolic pathway recruiting through genomic data analysis for industrial application of *Saccharomyces cerevisiae*. Biochem Eng J.

[126] Qiao Y, Li C, Lu X, Zong H, Zhuge B. Transporter engineering promotes the co-utilization of glucose and xylose by Candida glycerinogenes for D-xylonate production. Biochem Eng J. 2021;175.

[127] Chu J, Zhang S, Zhuang Y (2003). Fermentation process optimization of recombinant *Saccharomyces cerevisiae* for the production of human interferon-α2a. Appl Biochem Biotechnol.

[128] Payne T, Finnis C, Evans LR, Mead DJ, Avery SV, Archer DB, Sleep D (2008). Modulation of chaperone gene expression in mutagenized *Saccharomyces cerevisiae* strains developed for recombinant human albumin production results in increased production of multiple heterologous proteins. Appl Environ Microbiol.

[129] Favaro L, Cagnin L, Corte L, Roscini L, De Pascale F, Treu L, Campanaro S, Basaglia M, van Zyl WH, Casella S, Cardinali G. Metabolomic alterations do not induce metabolic burden in the industrial yeast M2n[pBKD2-Pccbgl1]-C1 engineered by multiple δ-integration of a fungal β-glucosidase gene. Front Bioeng Biotechnol. 2019;7. 10.3389/fbioe.2019.00376 PMC6893308 31850332

[130] Martínez JL, Meza E, Petranovic D, Nielsen J (2016). The impact of respiration and oxidative stress response on recombinant α-amylase production by *Saccharomyces cerevisiae*. Metabolic Eng Commun.

[131] Chen X, Li X, Ji B, Wang Y, Ishchuk OP, Vorontsov E, Petranovic D, Siewers V, Engqvist MKM (2022). Suppressors of amyloid-β toxicity improve recombinant protein production in yeast by reducing oxidative stress and tuning cellular metabolism. Metab Eng.

[132] Ishchuk OP, Domenzain I, Sánchez BJ, Muñiz-Paredes F, Martínez JL, Nielsen J, Petranovic D. Genome-scale modeling drives 70-fold improvement of intracellular heme production in *Saccharomyces cerevisiae*. PNAS*.* 2022;119:119. 10.1073/pnas.2108245119 PMC9335255 35858410

[133] Li F, Chen Y, Qi Q, Wang Y, Yuan L, Huang M, Elsemman IE, Feizi A, Kerkhoven EJ, Nielsen J (2022). Improving recombinant protein production by yeast through genome-scale modeling using proteome constraints. Nat Commun.

[134] Wang G, Björk SM, Huang M, Liu Q, Campbell K, Nielsen J, Joensson HN, Petranovic D. RNAi expression tuning, microfluidic screening, and genome recombineering for improved protein production in *Saccharomyces cerevisiae*.PNAS*.* 2019;116:9324–9332. 10.1073/pnas.1820561116 PMC6511059 31000602

[135] Huang M, Bao J, Hallström BM, Petranovic D, Nielsen J (2017). Efficient protein production by yeast requires global tuning of metabolism. Nat Commun.

[136] Huang M, Bai Y, Sjostrom SL, Hallström BM, Liu Z, Petranovic D, Uhlén M, Joensson HN, Andersson-Svahn H, Nielsen J. Microfluidic screening and whole-genome sequencing identifies mutations associated with improved protein secretion by yeast. PNAS*.* 2015;112:4689–4696. 10.1073/pnas.1506460112 PMC4553813 26261321

[137] Wang C, Zhang W, Tian R, Zhang J, Zhang L, Deng Z, Lv X, Li J, Liu L, Du G, Liu Y (2022). Model-driven design of synthetic N-terminal coding sequences for regulating gene expression in yeast and bacteria. Biotechnol J.

[138] Yu R, Nielsen J (2019). Big data in yeast systems biology. FEMS Yeast Res.

[139] Zelezniak A, Vowinckel J, Capuano F, Messner CB, Demichev V, Polowsky N, Mülleder M, Kamrad S, Klaus B, Keller MA, Ralser M (2018). Machine learning predicts the yeast metabolome from the quantitative proteome of kinase knockouts. Cell Syst.

[140] Liu J, Yang J, Yuan L, Wu C, Jiang Y, Zhuang W, Ying H, Yang S (2023). Modulated arabinose uptake and cAMP signaling synergistically improve glucose and arabinose consumption in recombinant yeast. J Agric Food Chem.

[141] Dixit Y, Yadav P, Sharma AK, Pandey P, Kuila A (2023). Multiplex genome editing to construct cellulase engineered *Saccharomyces cerevisiae* for ethanol production from cellulosic biomass. Renew Sustain Energy Rev.

